# Evidence from a Series of 104 Equine Sarcoids Suggests That Most Sarcoids in New Zealand Are Caused by Bovine Papillomavirus Type 2, although Both BPV1 and BPV2 DNA Are Detectable in around 10% of Sarcoids

**DOI:** 10.3390/ani11113093

**Published:** 2021-10-29

**Authors:** John S. Munday, Geoff Orbell, Rob A. Fairley, Michael Hardcastle, Bernie Vaatstra

**Affiliations:** 1 School of Veterinary Science, Massey University, Palmerston North 4410, New Zealand; 2 Gribbles Veterinary Ltd., Palmerston North 4410, New Zealand; Geoff.Orbell@gribbles.co.nz (G.O.); Bernie.Vaatstra@gribbles.co.nz (B.V.); 3 Gribbles Veterinary Ltd., Christchurch 8140, New Zealand; Rob.Fairley@gribbles.co.nz; 4 Gribbles Veterinary Ltd., Auckland 1060, New Zealand; Michael.Hardcastle@gribbles.co.nz

**Keywords:** horse, sarcoid, bovine papillomavirus, vaccine, New Zealand, viral trends, viral carcinogenesis, neoplasia, skin, *deltapapillomavirus*

## Abstract

**Simple Summary:**

Equine sarcoids are common cancers of horses that are caused by bovine papillomaviruses (BPVs). Previous studies have suggested that most sarcoids are caused by either BPV1 or BPV2, with the proportion caused by each BPV type dependent on the country in which the horse lived. Additionally, some studies also suggest that other papillomavirus (PV) types could cause equine sarcoids. The study was comprised of 104 sarcoids from New Zealand horses and used both specific and consensus PCR primer sets. Overall, PV DNA was amplified from 90% of the sarcoids. Of the ones that contained BPV DNA, 88% contained only BPV2 DNA, 10% BPV1 and BPV2 DNA, and 2% only BPV1 DNA. Only the primers specific for BPV1 or specific for BPV2 amplified DNA and no other PV types were detected. There was little variability in the rates of detection between different regions of New Zealand and rates were consistent when two distinct time periods were compared. These results suggest that sarcoids from New Zealand horses are consistently most often caused by BPV2 and thus if vaccination is used to prevent these cancers, it will be important to use a vaccine that provides good protection against this BPV type.

**Abstract:**

Equine sarcoids are common mesenchymal neoplasms of horses that are caused by cross-species infection by *deltapapillomaviruses*. While bovine papillomavirus (BPV) 1 and 2 are the most common causes, there are differences between countries regarding which of these BPV types cause the majority of sarcoids. Additionally, no causative PV can be detected in a subset of sarcoids, suggesting that other PV types could be rarer causes of these neoplasms. In the present study, consensus PCR primers and PCR primers specific for the five *deltapapillomavirus* types currently known to cause mesenchymal neoplasia (BPV1, BPV2, BPV13, BPV14, and Ovis aries PV2 DNA) were used to investigate the presence of PV DNA in 104 sarcoids from three defined regions in New Zealand and from two distinct time periods separated by 15 years. PV DNA was detected in 94 (90.4%) sarcoids. Of the sarcoids containing PV DNA, 83 (88.3%) contained only BPV2 DNA, 9 (9.6%) BPV1 and BPV2 DNA, and 2 (2.1%) only BPV1 DNA. No other PV types were detected. The predominance of BPV2 is consistent with studies of sarcoids from North America but dissimilar to studies of sarcoids from Europe and Australia. Detection rates of BPV1 and BPV2 were similar in sarcoids from different regions of New Zealand and in sarcoids from different time periods. These results suggest that most equine sarcoids in New Zealand are caused by BPV2 and thus if vaccines are developed to prevent sarcoids, vaccines that provide good protection against BPV2 will be required in this country.

## 1. Introduction

Sarcoids are the most common skin neoplasm of horses [1]. These mesenchymal neoplasms are typically locally invasive, although they do not metastasize [1]. Due to their invasive behavior and frequent recurrence after excision, sarcoids are a significant cause of morbidity in horses and globally are a significant concern in veterinary medicine [2]. 

Papillomaviruses (PVs) are double-stranded circular DNA viruses that are typically highly species-specific [3]. However, PVs in the *deltapapillomavirus* genus are unique in their ability to cause cross-species infections. In addition to being the only PV genus that can infect multiple hosts, five *deltapapillomavirus* types have been shown to cause mesenchymal tumors in their non-definitive host species. These are bovine PV (BPV) 1, BPV2, BPV13, BPV14, and Ovis aries PV (OaPV) 2 [4,5,6,7]. All the BPVs that have been shown to cause mesenchymal neoplasia are classified as species 4 *deltapapillomaviruses*, while OaPV2 is classified as a species 3 *deltapapillomavirus* [6,8]. 

Current evidence suggests that most equine sarcoids throughout the world contain BPV1 or BPV2 DNA [4]. These PVs are generally considered to be the cause of sarcoids. However, as BPV DNA has also been detected within the skin of normal horses, the pathogenesis of these lesions is not fully resolved and other factors in addition to the simple presence of the PV appear to be required for sarcoid development [9,10]. Interestingly, the proportion of sarcoids containing BPV1 and BPV2 varies markedly between different countries [11,12,13,14,15,16,17]. Although differences between countries are well recognized, few studies have determined whether differences in the predominant BPV type exist between regions within a country. Additionally, there have been no studies investigating whether the proportion of sarcoids caused by BPV1 and BPV2 in a region remains stable or changes over time. 

While BPV1 and BPV2 are thought to be the predominant causes of sarcoids, a proportion of sarcoids in some studies do not contain either BPV1 or BPV2 DNA [11,16]. This suggests the possibility that some sarcoids could be caused by a PV type that is neither BPV1 nor BPV2. The potential of other PV types to cause sarcoids was illustrated by the recent detection of BPV13 in an equine sarcoid from Brazil [5]. While BPV13 was not detected in a series of equine sarcoids from Europe [18], this PV type has been detected in samples from cattle in Italy and China, and thus could cause equine sarcoids more widely throughout the world [19,20]. Neither BPV14 nor OaPV2 have been reported to infect horses. However, as both these types have been shown to cause mesenchymal neoplasia after cross-species infection [6,7,21], both PV types could potentially be the cause of the sarcoids that contain neither BPV1 nor BPV2 DNA. 

Identifying the cause of equine sarcoids is important due to the development of virus-like particle (VLP) vaccines to prevent the development of these common neoplasms [22]. Vaccines containing BPV1 VLPs were found to stimulate high antibody titers and protect against pseudo-sarcoid formation after experimental inoculation with BPV1 [23,24]. However, there is some uncertainty from studies of human VLP vaccines about how well a VLP vaccine against one PV type protects against infection from other PV types [25,26]. In horses, BPV1 and BPV2 are very closely related serotypes. As in vitro studies showed that antibodies against either BPV1 or BPV2 reacted against both the homologous and heterologous BPV type, good cross-protection appears to be likely [27]. However, a VLP vaccine containing BPV1 and EcPV2 only appeared to show partial protection against infection by BPV2, possibly due to low antibody titers against BPV1 generated by the combined vaccine [24]. If a BPV1 VLP does not confer complete protection against infection by other BPV types, such a vaccine would be expected to be highly effective in a geographical region in which almost all sarcoids are caused by BPV1. However, if a significant number of sarcoids are caused by BPV2 or a different *deltapapillomavirus* type, it is possible that a BPV1 VLP vaccine may prevent a smaller proportion of sarcoids. 

The first aim of the reported study was to use specific PCR primers that amplify either BPV1 or BPV2 DNA to determine which of these PV types more commonly causes equine sarcoids in New Zealand. This is the first investigation of the causes of sarcoids in this country. In addition, the causative BPV types were compared within groups of sarcoids from different regions of New Zealand. Furthermore, sarcoids from two distinct time periods that were separated by 15 years were compared to determine whether the proportion of sarcoids caused by BPV1 and BPV2 remained constant over time. The second aim of the reported study was to use specific primers to determine if BPV13, BPV14, or OaPV2 DNA was present within the equine sarcoids. This is the first time that specific primers against all five different *deltapapillomavirus* types currently known to cause mesenchymal tumors have been used to detect PV DNA within equine sarcoids. 

## 2. Materials and Methods

### 2.1. Samples and DNA Extraction

**Figure 1:**
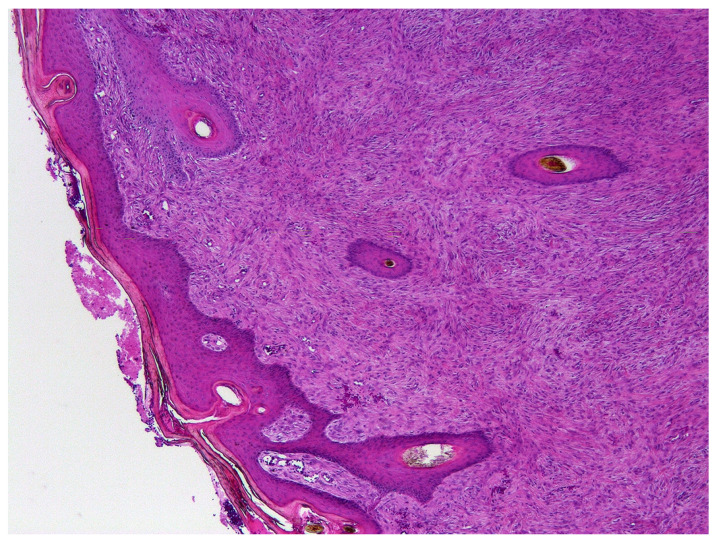
Photomicrograph of an equine sarcoid. Note the characteristic epidermal hyperplasia with the formation of rete pegs extending into a proliferation of mesenchymal cells arranged in tight whorls or bundles. HE 100×.

Paraffin tissue blocks containing formalin-fixed samples of equine sarcoids were identified by searching the databases of the Pathology Department at Massey University and Gribbles Veterinary Ltd, New Zealand. Samples were subclassified by the region of New Zealand that the sample was submitted from. Sarcoids from one region were also grouped into those submitted between 1996 and 2000, and those submitted between 2015 and 2020. The initial histological diagnosis was used to identify equine sarcoids. A hematoxylin and eosin-stained slide was subsequently examined by a veterinary pathologist boarded by the American College of Veterinary Pathologists to confirm this diagnosis (Figure 1). 

A 10 um shaving was taken from each block for DNA extraction. Careful positioning and cleaning of the microtome blade was done to ensure no cross-contamination between samples. Total DNA was extracted from each shaving using a NucleoSpin DNA FFPE XS kit (Macherey-Nagel GmbH, Duren, Germany) according to manufacturers’ instructions. When a case had multiple tissue blocks, a shaving was only taken from one so that if a horse had multiple sarcoids submitted, only one of these sarcoids would have been evaluated for the presence of PV DNA.

### 2.2. Amplification and Sequencing

The presence of amplifiable DNA within each sample was confirmed by amplifying part of the equine beta actin gene as previously described [28]. Five sets of specific primers (Table 1) were used as previously described [29]. Briefly, amplification conditions were 95 °C for 15 min, 45 cycles of 95 °C for 1 min, 60 °C for 30 s, and 72 °C for 1.5 min, with a final extension at 72 °C for 5 min. The positive controls used for the BPV1 and BPV2 primer sets was DNA from an equine sarcoid previously found to contain BPV1 DNA and DNA extracted from bovine anal fibropapilloma previous found to contain BPV2 DNA, respectively [30]. The positive controls for primers specific for BPV14 and OaPV2 were DNA extracted from a feline sarcoid and a sarcoid-like mass from the mouth of a pig, respectively [7]. Due to freight disruptions caused by COVID-19, it was not possible to import a sample containing BPV13 into New Zealand and no positive control was available for this primer set. The specificity of each primer set for the target *deltapapillomavirus* type has been previously confirmed [29]. 

In addition to the specific primer sets used in this study, three sets of consensus PCR primers were also used to amplify DNA from the sarcoids. These primers were only used to investigate the presence of PV DNA in sarcoids that did not contain PV DNA amplifiable by the specific PCR primer sets. The amplification conditions of the FAP59/64, MY09/11, and CP4/5 primer sets were as previously described [31]. Positive controls used for the consensus primers were DNA extracted from a canine cutaneous papilloma that contained canine papillomavirus type 1 DNA for the FAP59/64 primers and DNA extracted from a bovine anal fibropapilloma that contained BPV2 DNA for the MY09/11 and CP4/5 primers. A negative control that did not contain that template DNA was included with all PCR reactions. 

To further confirm that the specific primers were amplifying DNA from the expected PV type, 5 amplicons amplified by the jmBPV1 primers and 5 amplicons amplified by the jmBPV2 primers were purified and sequenced as previously described [32]. Results were compared with known sequences from GenBank (see http://www.ncbi.nlm.nih.gov/GenBank, accessed on 1 May 2021) using the basic local alignment search tool (http://www.ncbi.nlm.nih.gov/blast, accessed on 1 May 2021).

## 3. Results

A search of the databases allowed for the inclusion of 104 equine sarcoids, each from a different horse. Fourteen sarcoids were submitted from veterinary practices located in the upper North Island (UNI) of New Zealand, 45 were from practices within the lower North Island (LNI), and 45 were from practices in the South Island (SI). All the sarcoids from the UNI and SI were submitted between 2015 and 2020. However, the sarcoids that were submitted from practices in the LNI were further divided into 22 that were submitted between 1996 and 2000, and 23 that were submitted between 2015 and 2020. 

All 104 samples contained amplifiable beta actin DNA. Papillomaviral DNA was amplified from 94 of the 104 (90.4%) samples using at least one of the five specific PCR primer sets (Table 2). DNA was not amplified from 10 (9.6%) samples by any of the specific primer sets. In a similar manner, PV DNA was not amplified by any of the three consensus primer sets from these 10 sarcoids. The equine sarcoids that were found to contain PV DNA included 13 of the 14 (92.9%) samples from the UNI, 41 of the 45 (91.1%) of the LNI sarcoids, and 40 of the 45 (88.9%) sarcoids from the SI. When the LNI sarcoids were grouped into the two time periods, PV DNA was detected in 20 of the 22 (90.9%) sarcoids submitted between 1996 and 2000, and in 21 of the 23 (91.3%) sarcoids submitted between 2015 and 2020. 

Only the primer sets specific for BPV1 and BPV2 amplified PV DNA sequences from the samples and no DNA was amplified using the specific primer sets for BPV13, BPV14, or OaPV2. Of the 94 sarcoids that contained PV DNA, 83 (88.3%) contained only BPV2 DNA, 9 (9.6%) contained both BPV1 and BPV2 DNA, and 2 (2.1%) contained only BPV1 DNA (Table 3). Within the different regions of New Zealand, 92.3% of the sarcoids from the UNI, 85.4% from the LNI, and 90% from the SI contained only BPV2 DNA. Mixed infections were not detected in sarcoids from the UNI but were detected in 12.2% of the sarcoids from the LNI and 10% of the sarcoids from the SI. Only BPV1 DNA was detected in 7.7% of the sarcoids from the UNI, 2.4% of the sarcoids from the LNI, and none of the sarcoids from the SI. When sarcoids from the LNI were separated into the two time periods, only BPV2 DNA was detected in 90% of the sarcoids submitted between 1996 and 2000, and in 81% of the sarcoids submitted between 2015 and 2020. Mixed infections were detected in 10% of the earlier series of sarcoids and in 14.3% of sarcoids in the later series. Only BPV1 was detected in one of the sarcoids, which was submitted between 2015 and 2020. 

Sequencing of the amplicons revealed that the sequences amplified by the specific primers were all 98–100% similar to BPV1 or BPV2 sequences contained in GenBank (MG977494.1 for BPV1 and MN304951.1 for BPV2). The small number of mismatches within the nucleotide sequences was considered as due to errors in sequencing rather than as evidence of different strains of PV. 

## 4. Discussion

Around 90% of the sarcoids from New Zealand horses that contained detectible PV DNA contained only BPV2 sequences. In contrast, few (2.1%) were found to contain only BPV1 DNA. This suggests that BPV2 is the predominant cause of equine sarcoids in New Zealand. These results are most similar to a study of sarcoids from Canadian horses. In this study, 81% of 74 sarcoids contained only BPV2 DNA while 19% contained only BPV1 DNA [16]. Similarly, 55% of 54 sarcoids from horses in the western United States contained only BPV2 DNA [13]. In contrast, studies of European horses revealed that the majority of sarcoids are caused by BPV1, with only BPV1 was detected in 88% of 99 sarcoids from Dutch horses [12], 95% of 58 sarcoids from Swiss horses [33], 100% of 10 sarcoids from British horses [13], and 95% of 21 sarcoids from horses in Poland [15]. In a similar manner, 70% of 10 sarcoids from Japanese horses contained only BPV1 DNA [17]. Interestingly, in a study of sarcoids from horses from Australia, the country most geographically close to New Zealand, 82% of 34 sarcoids contained only BPV1 DNA, while 18% contained only BPV2 DNA [11]. 

The results of the present study provide further evidence that the world can be subdivided into regions in which equine sarcoids are predominantly caused by BPV2 and regions in which sarcoids are predominantly caused by BPV1. Currently, North America and New Zealand comprise the regions in which most sarcoids are caused by BPV2, while most sarcoids in Europe, Japan, and Australia appear to be caused by BPV1. The reason for the differences in the predominant cause of equine sarcoids between countries is currently unknown. However, as BPVs have cattle as their definitive host, it is possible that the predominant cause of equine sarcoids in a country is determined by the predominant BPV type to infect cattle in that country. There are few studies investigating the common BPV types infecting cattle and such studies can be difficult to interpret as cattle are often infected with multiple BPV types [34]. However, BPV1 DNA, but not BPV2 DNA, was amplified from teat samples of Japanese cattle [35], potentially corresponding with the predominance of BPV1 in equine sarcoids in this country [17]. Furthermore, consistent with the predominance of BPV1 in European sarcoids, 60% of warts from German cattle contained BPV1 DNA, with BPV2 only detected in 25% [36]. While the reason for the geographical variability in the cause of equine sarcoids is currently unknown, determining why 90% of sarcoids from New Zealand horses appear to be caused by BPV2 while 82% of sarcoids from Australian horses appear to be caused by BPV1 may help to understand the pathogenesis of these common equine tumors. 

Around 10% of the sarcoids that contained PV DNA in the present study contained both BPV1 and BPV2 DNA sequences. This is the second time that mixed infections with BPV types have been detected in a sarcoid. Previously, 7% of sarcoids from horses in the western United States were found to contain both BPV1 and BPV2 DNA [13], while mixed infections were not detected in any of the studies of sarcoids from European, Australian, and Japanese horses [11,12,14,15,17,33]. It is possible that this difference is again due to regional differences in the cause of equine sarcoids. Alternatively, the detection of mixed infections in multiple sarcoids in the present report could be due to the use of different methods to amplify PV DNA from the tumors. Previous studies have either used primers to amplify both BPV1 and BPV2, with the amplified type determined by the restriction length polymorphism assay [11,12], or used consensus PCR primers with the PV type determined by sequencing of the PCR product [15]. It appears probable that neither of these methods would detect a mixed infection if one PV type is present at much higher concentrations than other types present in the sample. This is because it is most likely that differences in the concentration of DNA from each type would be multiplied during PCR amplification so that the presence of PVs present at lower concentrations would be masked. In contrast, the use of specific primers in the present study enables the detection of small quantities of multiple PV types within the samples, even if one PV type is present at much higher concentrations. To the authors’ knowledge, this is the first time specific primers to detect BPV1 and BPV2 have been used to detect BPV DNA in a large series of sarcoids. Additional studies using specific BPV1 and BPV2 primers to investigate sarcoids from other countries will determine whether the frequent mixed infections observed in the present study was due to the use of different methods or due to true regional variability. 

The detection of mixed infections by BPV1 and BPV2 within the sarcoids suggests that both BPV types could have contributed to sarcoid development. It is currently unknown how horses are infected by BPVs, with exposure to BPVs from environmental contamination due to transmission from other infected horses, or through the vectors proposed [37]. Although seemingly unlikely, it is possible that a horse could be simultaneously infected by both BPV1 and BPV2 by any of these mechanisms. Alternatively, it is possible that one of the detected BPV types caused the sarcoid, with the other BPV type present within the sarcoid as an incidental infection. The possibility of incidental infection by one of the types is supported by the previous detection of BPV DNA in the skin of horses without sarcoids [9,10,38]. Quantification of the amount of DNA from both BPV types within the mixed infections may allow for a better understanding of the role of the two BPV types within the sarcoids. 

Ten percent of the sarcoids in the present study did not contain any detectible BPV1 or BPV2 DNA despite equine beta actin DNA being amplified from the sample. As BPV13, BPV14, and OaPV2 are all *deltapapillomaviruses* that have been shown to cause mesenchymal neoplasia, specific primer sets were used to try to amplify DNA from these PV types. These primers were used both to detect PV DNA from the sarcoids that did not contain BPV1/BPV2 DNA but also to detect mixed infections in sarcoids that did contain BPV1 or BPV2. Consistent with the results of a recent study of sarcoids from European horses [18], none of the sarcoids from New Zealand horses contained amplifiable BPV13 DNA. This suggests that this PV type is not a significant cause of sarcoids in New Zealand. BPV13 was not amplified from a series of warts from New Zealand cattle and this PV type has never been detected in New Zealand [29]. Although BPV14 has been previously detected in New Zealand [21], this PV type was not amplified from any equine sarcoid. This suggests that, despite the close phylogenetic relationship of BPV14 to BPV1 and BPV2 [6], this PV is, at most, a rare cause of equine sarcoids. Although OaPV2 has also been detected in New Zealand [7], there was no evidence from the present study that this PV type is able to cause equine sarcoids. Consensus primers were also used to detect PV DNA in the samples that did not contain PV DNA amplifiable by any of the specific primers sets. This was done to detect any PV types other than BPV1, BPV2, BPV13, BPV14, and OaPV2 that could have been present. However, these primers did not amplify PV DNA from any of the sarcoids evaluated. As no non-*deltapapillomavirus* PV types have been previously detected in sarcoids, the failure to detect PV DNA in these samples was not unexpected. The absence of detectible BPV1/2 DNA in these samples could also be due to these samples being misdiagnosed as sarcoids. While a diagnosis of sarcoid was made and then confirmed by two experienced veterinary pathologists, sarcoids are difficult to differentiate from other mesenchymal neoplasms of the skin [39] and it remains possible that the tumors that did not contain PV DNA were misclassified as sarcoids. Alternatively, the absence of PV DNA in the 10 sarcoids may have been due to the use of formalin-fixed tissue. Formalin fixation causes breaks in the DNA, which can prevent subsequent amplification [40]. 

As the results of the present study suggest that most sarcoids in New Zealand are caused by BPV2, if a BPV1 VLP vaccine is found not to induce complete protection against BPV2, the use of a combined BPV1 and BPV2 VLP vaccine would appear to be required for the optimal protection against sarcoid development in this country. The results also suggest that a vaccine to prevent infection with BPV13, BPV14, and OaPV2 would not be beneficial to prevent sarcoids in New Zealand. 

There was little variability in the rates of detection of the different BPV types in sarcoids from different regions within New Zealand. This suggests that the factor that results in BPV2 being the predominant BPV type to cause sarcoids in New Zealand is present throughout the country. In a similar manner, similar rates of detection of BPV2 and BPV1 DNA were observed in sarcoids submitted from the same region, although from two different time periods separated by 15 years. This is the first time that the causes of sarcoids within two distinct time periods have been compared. The results showed that the predominant BPV cause of sarcoids remained consistent over 15 years. These results suggest that a vaccine designed to protect against sarcoids in New Zealand will be effective throughout all regions and is likely to continue to be effective at preventing infection from the predominant BPV type for at least 15 years. 

## 5. Conclusions

In conclusion, the results of this study show that BPV2 is the predominant cause of equine sarcoids in New Zealand. The etiology of sarcoids in New Zealand is more similar to that of sarcoids in North America than that of sarcoids in Europe or Australia. The predominance of BPV2 is consistent throughout New Zealand and has remained stable for the last 15 years. The detection of mixed infections in sarcoids was unexpected and was likely because this is the first time that specific primer sets to amplify BPV1 and BPV2 have been used in a large series of sarcoids. These results are important in determining strategies to prevent equine sarcoids and to provide additional clues regarding the epidemiology of infection of horses with BPVs. 

## Figures and Tables

**Table 1:** The primer sequences used to amplify the five *deltapapillomavirus* types in this study. BPV is bovine papillomavirus and OaPV is Ovis aries papillomavirus.

Papillomavirus DNA Amplified	Primer	Length of Amplicon (bp)
BPV1	jmBPV1F. 5’-AGCTGTGATTTCCACAGAGC-3’	111
	jmBPV1R. 5’-TGGAACCCCACTAACAGAGT-3’	
BPV2	jmBPV2F. 5’-CTGTGCCTCCTAGTGGTTGG-3’	198
	jmBPV2R. 5’-TACCAAGTCACTGTGGGGGA-3’	
BPV13	jmBPV13F. 5’- ACAGTTGAACATCCTGCCCC-3’	112
	jmBPV13R. 5’- ATCCCAAAACCGTAGCCCTG-3’	
BPV14	jmBPV14F. 5’-GGAACAAACCTCACAATCAC-3’	195
	jmBPV14R. 5’-CCAGTTCTCTAATACTGAGG-3’	
OaPV2	jmOaPV2F. 5’-CTCGTAACCATTGCCTCATGC-3’	219
	jmOaPV2R. 5’-TGCCAGCAACAATCAGGCTA-3’	

**Table 2:** Summary of regions and time periods from which the equine sarcoids were submitted. The number of sarcoids from each region and the time to contain detectible papillomaviral (PV) sequences is included.

Group	PV DNA (%)	No PV DNA (%)	Total
All sarcoids	94 (90.4)	10 (9.6)	104
Upper North Island	13 (92.9)	1 (7.1)	14
Central North Island	41 (91.1)	4 (8.9)	45
1996–2000	20 (90.9)	2 (9.1)	22
2015–2020	21 (91.3)	2 (8.7)	23
South Island	40 (88.9)	5 (11.1)	45

**Table 3:** Summary of the type of bovine papillomavirus (BPV) detected in the equine sarcoids. This table only includes those sarcoids that were found to contain papillomavirus DNA.

Group	Only BPV1 (%)	Only BPV2 (%)	BPV1 and BPV2 (%)	Total
All sarcoids	2 (2.1)	83 (88.3)	9 (9.6)	94
Upper North Island	1 (7.7)	12 (92.3)	0 (0)	13
Central North Island	1 (2.4)	35 (85.4)	5 (12.2)	41
1996–2000	0 (0)	18 (90)	2 (10)	20
2015–2020	1 (4.7)	17 (81)	3 (14.3)	21
South Island	0 (0)	36 (90)	4 (10)	40
